# High-Throughput Mutation Profiling of Primary and Metastatic Endometrial Cancers Identifies *KRAS*, *FGFR2* and *PIK3CA* to Be Frequently Mutated

**DOI:** 10.1371/journal.pone.0052795

**Published:** 2012-12-27

**Authors:** Camilla Krakstad, Even Birkeland, Danila Seidel, Kanthida Kusonmano, Kjell Petersen, Siv Mjøs, Erling A. Hoivik, Elisabeth Wik, Mari Kyllesø Halle, Anne M. Øyan, Karl-Henning Kalland, Henrica Maria Johanna Werner, Jone Trovik, Helga Salvesen

**Affiliations:** 1 Department of Clinical Medicine, University of Bergen, Bergen, Norway; 2 Department of Gynecology and Obstetrics, Haukeland University Hospital, Bergen, Norway; 3 Department of Translational Genomics, University of Cologne, Cologne, Germany; 4 Max Planck Institute for Neurological Research, Cologne, Germany; 5 Laboratory of Translational Cancer Genomics, Center of Integrated Oncology Köln – Bonn, University of Cologne, Cologne, Germany; 6 Computational Biology Unit, Uni Computing, Uni Research AS, Bergen, Norway; 7 The Gade Institute, University of Bergen, Bergen, Norway; 8 Department of Microbiology, Haukeland University Hospital, Bergen, Norway; Peter MacCallum Cancer Centre, Australia

## Abstract

**Background:**

Despite being the most common pelvic gynecologic malignancy in industrialized countries, no targeted therapies are available for patients with metastatic endometrial carcinoma. In order to improve treatment, underlying molecular characteristics of primary and metastatic disease must be explored.

**Methodology/Principal Findings:**

We utilized the mass spectrometric-based mutation detection technology OncoMap to define the types and frequency of point somatic mutations in endometrial cancer. 67 primary tumors, 15 metastases corresponding to 7 of the included primary tumors and 11 endometrial cancer cell lines were screened for point mutations in 28 known oncogenes. We found that 27 (40.3%) of 67 primary tumors harbored one or more mutations with no increase in metastatic lesions. *FGFR2*, *KRAS* and *PIK3CA* were consistently the most frequently mutated genes in primary tumors, metastatic lesions and cell lines.

**Conclusions/Significance:**

Our results emphasize the potential for targeting *FGFR2*, *KRAS* and *PIK3CA* mutations in endometrial cancer for development of novel therapeutic strategies.

## Introduction

Despite being the most common pelvic gynecologic malignancy in industrialized countries, no targeted therapies are available for patients with metastatic endometrial carcinoma. Although 75% are treated at an early stage, 15% to 20% recur. For patients with advanced disease at diagnosis or recurrent disease, outcome is poor. In order to improve treatment, underlying molecular characteristics of primary and metastatic disease must be explored. Furthermore, improved tools for correct stratification of patients according to risk-groups and improved definitions of potential targets for novel therapeutics are of great importance and much work is undertaken to develop better criteria to select patients for individualized therapies [1].

To assess the risk of recurrent disease, traditionally endometrial cancer has been divided into two subgroups, type I and type II carcinomas [2]. Type I endometrial carcinoma is associated with good prognosis, low grade, endometrioid histology and rarely metastasize to regional and distant sites [3]. In addition, type I endometrial cancers are often hormone receptor positive with *PTEN* and *KRAS* mutations. Type II endometrial carcinomas are associated with poor prognosis, non-endometrioid histology, high grade, loss of hormone receptors and altered expression of p53 and p16. Still, the value of this classification to predict prognosis and for treatment stratification is limited as 20% of type I endometrial cancers recur and 50% of type II cancers do not [4].

Currently, conventional chemotherapy regimens and anti-hormonal treatment are basis for adjuvant and systemic treatment of recurrent or metastatic endometrial cancer as targeted therapies are not yet available in the clinic. However, mutational profiles are applied for selection of targeted therapeutics for several other cancers and also applied for clinical trials stratification. Our previous screening of a smaller number of endometrial cancer patients identified somatic mutations in FGFR2, KRAS, PIK3CA, PTEN, PT53 and CTNNB1 [5]. However, this study did not rule out possible mutations in other known oncogenes that could be potentially interesting for targeted treatment of endometrial cancer. Thus, the current study was undertaken to screen for a large panel of known oncogenic mutations in a series of primary and metastatic lesions from endometrial cancer patients using the high-throughput method OncoMap [6], [7]. OncoMap provides a unique opportunity to simultaneously interrogate a large number of known mutations in a large number of genes, thus providing the opportunity to characterize the molecular subgroups of endometrial cancer with a potential relevance for targeting novel therapeutics.

## Methods

### Ethics statement

All parts of the study have been approved according to Norwegian legislation as well as international demands for ethical review. The study was approved by the Norwegian Data Inspectorate, Norwegian Social Sciences Data Services, and the Western Regional Committee for Medical and Health Research Ethics, REC West (NSD15501; REK 052.01). Patients were included in the study after written informed consent approved by the ethics committee (REK West).

### Specimens

We have studied a total of 69 patients for mutations in 28 known oncogenes (Table 1). 23 of the included patients had previously been screened for fewer oncogene mutations by another method [5]. The patients were recruited from a population based patient series of 701 patients with endometrial cancer prospectively collected at Haukeland University Hospital, Norway. Age at diagnosis, FIGO stage, histological subtype and grade, treatment and follow-up was registered as previously reported [8]. Distribution of clinico-pathologic variable for the 69 investigated cases did not differ significantly from the larger (n = 701) unselected patient cohort (Table 2). Tissue was available from 67 primary tumors and 15 metastatic lesions from 9 patients of which 7 had corresponding tissues from primary lesions available for comparison. The majority of selected lesions were verified by frozen sections to contain >80% malignant epithelial component with a minimum cut off for inclusion of 50% purity.

**Table 1:** List of genes with number of mutations (n) screened for in OncoMap^1^.

Gene	Mutations (n)
ABL1	13
AKT2	2
ALK	13
BRAF	29
CDK4	2
DDR2	10
EGFR	55
EPHA3	16
EPHA5	6
ERBB2	22
ERBB4	9
FGFR1	3
FGFR2	15
FGFR3	11
FGFR4	11
FLT3	5
HRAS	16
JAK2	1
KDR	8
KIT	42
KRAS	19
MDM2	1
NRAS	18
NTRK1	8
NTRK3	10
PDGFRA	20
PIK3CA	16
RET	6

1 Detailed information on gene mutations and nucleotide changes is given in Table S1.

**Table 2:** Clinico-pathologic characteristics of 69 endometrial cancer patients screened in OncoMap compared to the whole population from the same region.

Variable	OncoMap n (%) Total n = 69*	Whole population n (%) Total n = 701§
Age, median	65	65
Menopause		
Pre-/Peri-	13 (19)	87 (12)
Post-	56 (81)	614 (88)
FIGO-09 stage		
I–II	56 (81)	577 (82)
III–IV	13 (19)	124 (18)
Histologic type		
Endometrioid	58 (84)	551 (79)
Non-endometrioid	11 (16)	150 (21)
Histologic grade		
Grade 1/2	46 (68)	449 (65)
Grade 3	22 (32)	243 (35)
Metastatic nodes		
Negative	38 (83)	484 (88)
Positive	8 (17)	64 (12)
ERα		
Positive	49 (75)	365 (77)
Negative	16 (25)	111 (23)

* Missing (n = 69); Grade: 1, Metastatic nodes: 23, ERα: 4.

§ Missing (whole population); Grade: 9, Metastatic nodes: 153, ERα: 225.

### Cell lines

Endometrial cancer cell lines Hec1A, Hec1B, KLE, RL95-2, ECC1 were purchased from ATCC-LGC Standards, London, UK, MFE-280, MFE-296, MFE3-19, EFE-184, AN3-CA were from DSMZ, Germany and Ishikawa from Sigma-Aldrich, St.Louis, MO. All cells were maintained in medium as recommended by the supplier, supplemented with Penicillin/Streptomycin (Sigma-Aldrich, St.Louis, MO).

### OncoMap and DNA sequencing

DNA from primary and metastatic lesions was extracted from fresh frozen biopsies. DNA was isolated by digestion over night at 65°C in lysis buffer containing proteinase K, followed by a standard ethanol precipitation. DNA from 11 endometrial cancer cell lines was extracted using Qiagen Tissue DNA kit according to manufacturers protocol. DNA quantity was measured using the Quant-iT™ Picogreen® Assay (Invitrogen) and high quality of the DNA assured on a 0.7% agarose gel before genomic DNA was amplified using the Repli-g Midi Kit (Qiagen, Germany) according to manufacturers' instructions. Amplified DNA was diluted 1∶10 in 1xTE buffer (pH 8.0) and after hydration for 24 h at room temperature further diluted to a working concentration of 5 ng/µl in water. Mutations were detected in genome-amplified DNA using a mass spectrometry-based single base extension technique (Sequenom, Inc.) as previously described [7]. Primers for additional assays to detect mutations described in several cancer studies since 2008 [9], [10], [11], [12], [13] were designed using the Sequenom Assay Design Software. Following amplification and mutation site specific probe elongation analytes were spotted on SpectroCHIPs I and masses detected using a Bruker matrix-assisted laser desorption/ionization–time of flight mass spectrometer (Sequenom). Spectra were manually reviewed using the Typer 4.0 Software (Sequenom). A list of the mutations included in OncoMap and the corresponding amino acid changes is given in Table S1.

To validate the proportion of the most frequently mutated oncogenes detected by OncoMap, genomic DNA was extracted from freshly frozen primary tumor tissue from 199 additional patients. In total 264 patients were screened for point mutations in *KRAS* (exon 2) and *PIK3CA* (exon 9 and 20) as described [14]. Details regarding primers and conditions are available upon request. Sequencing reactions were analyzed on an ABI Prism 3100 genetic analyzer using the Sequencing Analysis software, version 3.7.

### Oligonucleotide DNA microarray analyses

A microarray dataset corresponding to the 69 primary tumor samples included in the OncoMap screen was generated. RNA was extracted using the RNeasy Mini Kit (Qiagen, Hilden, Germany) and hybridized to Agilent Whole Human Genome Microarrays 44k (Cat.no. G4112F), according to the manufacturers instructions. Arrays were scanned using the Agilent Microarray Scanner Bundle and data were imported and analyzed in J-Express software (Molmine, Norway). Median spot signal was used as intensity measure. Expression data were normalized using quantile normalization. Microarraydata have been deposited in the ArrayExpress Archive database, http://www.ebi.ac.uk/arrayexpress/ (ArrayExpress accession: E-MTAB-1358).

A SAM (Significance Analysis of Microarray) analysis between grade I–II and grade III was performed to identify significantly differentially expressed genes according to histologic grade. 306 genes were significantly differentially expressed (FDR<0.01) between the two groups. Hierarchical clustering was performed on this list of genes using weighted average linkage and Pearson correlation as similarity measures. Clinico-pathological data and mutational status were mapped manually to the cluster-tree to visualize the distribution of mutation across the patient population.

## Results

The OncoMap screen for 387 oncogenic mutations in 28 commonly mutated genes in cancer (Table S1) was applied in 67 primary and 15 metastatic endometrial carcinoma lesions as well as 11 endometrial carcinoma cell lines and detected mutations in 7 of the investigated genes. We found that 27 patients (40.3%) had point mutations in one single gene, while 4 patients (6.0%) had mutations in 2 genes. Among the seven genes with detected somatic mutations in primary and metastatic lesions, *KRAS* (17.9%), *PIK3CA* (14.6%) and *FGFR2* (10.4%) were the most frequently mutated, while mutations in *BRAF* (1.5%), *EGFR* (1.5%), *HRAS* (1.5%) and *NRAS* (1.5%) were rare. The frequencies of *KRAS* and *PIK3CA* mutations were validated by DNA sequencing in 264 primary tumors (Table 3). *FGFR2* mutation frequency had been validated previously [5]. The most common single mutation found by OncoMap screening was *FGFR2* aaS252W (9.0%), however the most frequently mutated gene was *KRAS* (17.4%) (Table 3). The OncoMap screen of the 11 established endometrial cancer cell lines identified as expected *KRAS* G12D and *PIK3CA* G1049R mutations in both Hec1A and Hec1B, while *FGFR2* mutation S252W was found in MFE280 and MFE319. Additionally, two PIK3CA mutations were identified in MFE280 and MFE296 (E545K and P539R, respectively). We did not find any of the cell lines to have mutations in any of the other genes included in the OncoMap panel.

**Table 3:** Frequency of mutations in 67^1^ primary lesions from endometrial cancer patients.

Gene	aa	OncoMap n = 67^2^		Validated n = 264 (%)
		n	(%)	
*FGFR2*	S252W	6	9	
	P253R	1	1.5	
***Total:***		***7***	***10.4***	*12.3* [5] 3
*KRAS*	G12C	3	4.5	
	G13D	3	4.5	
	G12D	3	4.5	
	G12A	1	1.5	
	*total Exon 2*	*10*	*16.1*	*14.7*
	Q61H	2	3.0	
***Total:***		***12***	***17.9***	
*PIK3CA*	R88Q	2	3.0	
	Q546K	2	3.0	
	E545K	2	3.0	
	P539R	1	1.5	
	*total Exon 9*	*7*	*7.5*	*5.8*
	M1043I	1	1.5	
	H1047R	1	1.5	
	*total Exon 20*	*2*	*3.2*	*8.8*
***Total:***		***9***	***11.9***	*14.6*
*BRAF*	F468C	1	1.5	
*EGFR*	T790M	1	1.5	
*HRAS*	G125	1	1.5	
*NRAS*	Q61L	1	1.5	

1 data missing for 2 primary tumors, n: number of mutated samples.

2 23 of the samples previously subjected to DNA sequencing of all exons of 89 tyrosine kinase genes and 19 additional known oncogenes and tumor suppressor genes as reported [5].

3 Validated in a dataset independent of the present study.

**Figure 1:**
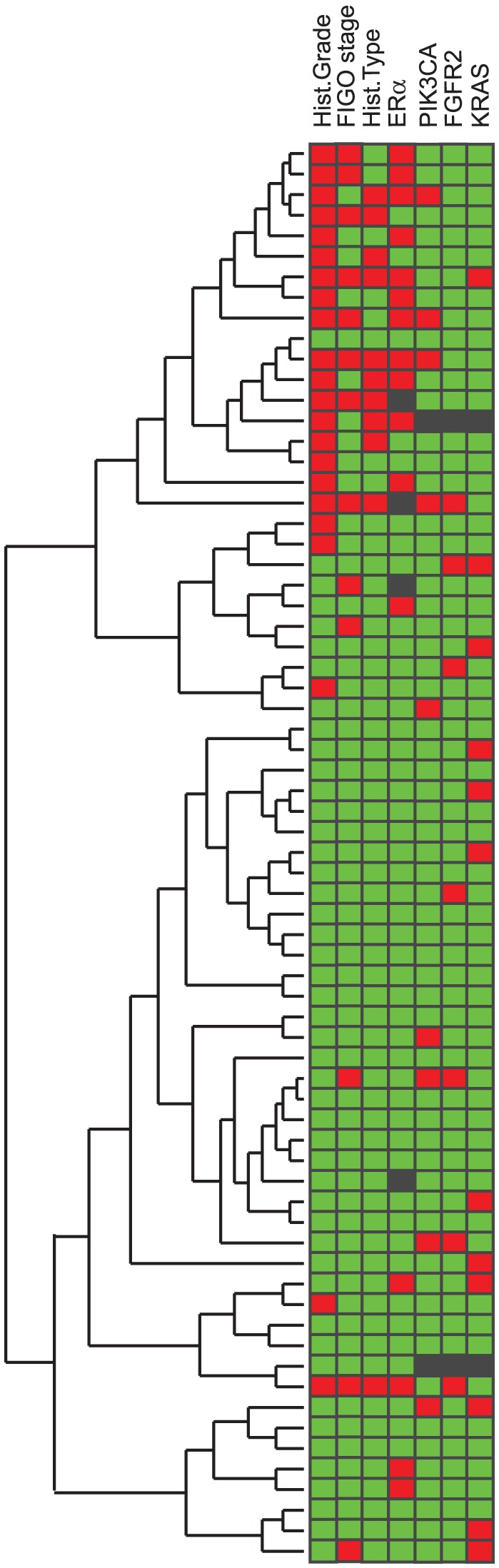
Mutational status is not reflected in distinct patient clusters related to phenotype. A hierarchical clustering of 306 significantly differentially expressed genes between grade I–II and grade III was mapped with clinico-pathological data and mutational status to visualize the distribution of mutation across the patient population. Green square color indicate good prognosis groups (Grade I–II, FIGO I–II, endometrioid type, ERα positivity) and no detected mutation in indicated gene, Red square color indicate poor prognosis groups (Grade III, FIGO III–IV, non-endometrioid types, ERα negativity) and detected mutation in indicated gene. Black square: data missing.

To explore a possible link between type of mutations and gene expression patterns in primary tumors, a hierarchical cluster analysis of 306 genes significantly differentially expressed (SAM analysis, FDR<0.01) according to histologic grade was performed. We found that there was no significant association between specific oncogene mutations and patient clusters based on transcriptional signatures (Figure 1). This finding appears to be in line with our previous report on a smaller data set applying an earlier generation of mRNA genearrays, with no enrichment for PIK3CA mutations in the patient cluster capturing aggressive phenotype [15].

To further investigate if mutation pattern changed during disease progression, 15 metastatic lesions from 9 patients from which seven had primary tumors available for comparison, were analyzed for mutations. *KRAS*, *PIK3CA* and *FGFR2* were found to be the most frequently mutated genes also in metastatic lesions, with no significant increase in mutation frequency (Table 4). In two cases, mutations were detected in the metastatic lesions but not in the primary lesion, while one case with mutation in the primary lesion had no detectable mutation in the metastatic lesion. The small sample set available for this analysis, tumor heterogeneity and differences in stromal contamination should call for caution in the conclusions.

**Table 4:** Mutational status in primary endometrial cancers and corresponding metastatic lesions.

	Primary Tumors		Corresponding metastatic lesions			
ID	Gene	AA	Met ID	Gene	AA	Site of met
499	n.m.d^1^		499a	*PIK3CA*	R88Q	Spleen
394	n.m.d		394a	n.m.d		Vagina
1749	Data missing	1749a	n.m.d		Lymph node	
			1749b	n.m.d		Lymph node
			1749c	n.m.d		Lymph node
492	Data missing	492a	*PIK3CA*	E545K	Oment	
			492b	*PIK3CA*	E545K	Gastric
279	*PIK3CA*	P539R	279a	n.m.d		Oment
1393	*PIK3CA*	R88Q	1393a	*PIK3CA*	R88Q	Cervix
1406	*PIK3CA FGFR2*	E545K S252W	1406a	*FGFR2*	S252W	Cervix
			1406b	*FGFR2*	S252W	Vagina
				*PIK3CA*	E545K	
621	*FGFR2*	S252W	621a	n.m.d		Parametrium
1495	*KRAS*	G12D	1495a	*KRAS*	G12D	Vagina
			1495b	*KRAS*	G12D	Ovary
			1495c	*KRAS*	G12D	Ovary

1 n.m.d: no mutation detected.

## Discussion

Activating mutations in specific proto-oncogenes may confer oncogene-addiction. Such mutations have been identified in several genes and may drive malignant disease progression. This principal for oncogene-addiction can be exploited to develop new targeted therapies [16]. Currently, mutational profiles are applied for selection of targeted therapeutics for e.g. BRAF inhibitors in malignant melanoma [17] and BRAF and EGFR targeting in lung- and colorectal cancers [18], [19]. For endometrial cancer, none of the novel targeted therapeutics is available in the clinic at present. However, several ongoing clinical trials aim at exploiting targets supported by recent comprehensive molecular profiling of primary endometrial carcinoma lesion [1], dominated by trials targeting the phosphatidylinositol 3-kinase (PI3K)/AKT/mammalian target of rapamycin (mTOR) or FGFR2. However, to our knowledge, no previous study has reported as comprehensive mutational data for a large panel of oncogenes in endometrial cancers including metastatic lesions.

A large number of oncogene mutations has been identified to be important in cancer development. Recently, several papers have reported the usefulness of the high-throughput genotyping platform OncoMap to screen for mutations in a large panel of known cancer oncogenes [6], [20], [21], [22]. The high degree of concordance between our findings using OncoMap for the investigated genes and the validated frequency in the present study as well as previously published mutation frequencies in endometrial cancer samples based on traditional sequencing, is assuring. Using OncoMap we found that 40.3% of the analyzed endometrial cancer samples harbored at least one mutation. Of the 28 oncogenes included, mutations were only found at high frequency (>10%) in *KRAS*, *PIK3CA* and *FGFR2*. These genes have been linked to endometrial cancer previously, both by us [5], [15] and others [23], [24].

In the present study, the S252W mutation in *FGFR2* was identified as the most frequent single mutation (9%) in endometrial cancer. The somatic *FGFR2* mutations include the S252W and P253R alleles, where autosomal dominant mutations are associated with the congenital developmental disorder Apert syndrome [25]. We, and others [26], have previously linked these mutations to endometrial cancer, through increased tumor cell survival and anchoring independent growth in endometrial cancer cell lines, and indicated the potential for FGFR2 inhibitors in mutated cell lines [5]. It has also been reported that FGFR2 inhibitors induce cell death in endometrial cancer cells despite *PTEN* inactivating mutations [27]. The frequency of *FGFR2* mutations detected in the present OncoMap screen of 10.4% is in concordance with our previous findings from 122 endometrial cancer patients from the same region, finding *FGFR2* to be mutated in 12.3% [5]. Recently, a frequency in this range of 10.3% was also published by others [23].

Several of the *PIK3CA* mutations were detected at relatively low frequencies (<3%), however the total frequency of any detected *PIK3CA* mutations was 13.4%. We have validated this frequency of point mutations in *PIK3CA* (exon 9 and 20) in 14.6% in a cohort of 264 endometrial cancer patients. This is consistent with the reported mutational frequency of *PIK3CA* in endometrial carcinoma in the COSMIC database for *PIK3CA* mutations tested for in OncoMap [28]. A potential relevance for targeting therapy in patients harboring *PIK3CA* mutations was recently supported in a study demonstrating higher response rate to PI3K/AKT/mTOR inhibitors for patients with mutated compared to wild type *PIK3CA* in breast and gynecologic malignancies [29].

*KRAS* mutations were found in 17.9% of the cases, with high frequency of point mutations in exon 2 (G12A, G12C, G12D and G13D), validated in 264 endometrial cancer patients (14,7%; [14]) and also in line with previous studies (18%; [30]). *KRAS* mutations have been associated with low grade, and endometrioid histologic subtype, although not with prognosis [31], [32]. Interestingly, *KRAS* and *FGFR2* mutations were found to be mutually exclusive, in line with a previous report [23]. In terms of therapy KRAS mutational status has been linked to EGFR inhibitor resistance in colorectal cancer [33], but further studies are needed in endometrial carcinoma to explore such potential link.

In line with the present study, we previously reported a low frequency (2%) of mutations in *BRAF* in endometrial cancer [30]. Interestingly, with the exception of a few mutations in *NRAS*, *HRAS*, *EGFR* and *BRAF* (1.5%), no other hot-spot mutations were identified in the remaining 21 oncogenes screened for, neither in primary tumors nor in metastatic lesions.

The present work used a version of OncoMap covering 387 mutations in a total of 28 different oncogenes. In endometrial carcinomas, the oncogenes *CTNNB1* and tumor suppressor genes *PTEN* and *P53* have also been reported to be frequently mutated [34], [35] but were not included in the present sceen and can therefore not be accounted for. Among the included genes and mutations, we have identified and validated *KRAS*, *PIK3CA* and *FGFR2* to be the most frequently mutated oncogenes in endometrial cancer. Although transcriptional signature pattern according to histologic grade did not identify any distinct subgroups linking any of the mutations to phenotype, *PIK3CA*, *KRAS* and *FGFR2* mutations may still be of relevance for targeting novel therapeutics in endometrial cancer. Nevertheless, more knowledge regarding functional aspects of the different mutations and their implications for response to drugs will be important to guide further selection of patients for molecularly based clinical trials.

## Supporting Information

Table S1 **Oncogene mutations and nucleotide changes included in OncoMap.**
(DOCX)
