# Supplementary material for: High-Throughput Mutation Profiling of Primary and Metastatic Endometrial Cancers Identifies KRAS, FGFR2 and PIK3CA to Be Frequently Mutated
Source: PLoS One. 2012 Dec 27;7(12):e52795. doi: 10.1371/journal.pone.0052795 (PMC3531332; doi:10.1371/journal.pone.0052795)
Supplement: Table S1 — Oncogene mutations and nucleotide changes included in OncoMap. (DOCX) [file pone.0052795.s001.docx]

Table S1. Oncogene mutations and nucleotide changes included in OncoMap

| **Gene** | **Amino Acid_change** | **Nucleotide_change** |
| --- | --- | --- |
| ABL1 | G250E | 749G>A |
|  | Q252H | 756G>C |
|  | Y253H | 757T>C |
|  | Y253F | 758A>T |
|  | E255K | 763G>A |
|  | E255V | 764A>T |
|  | T315I | 944C>T |
|  | F317L | 951C>G |
|  | M351T | 1052T>C |
|  | E355G | 1064A>G |
|  | F359V | 1075T>G |
|  | H396R | 1187A>G |
|  | F311L | 931T>C |
| AKT2 | S302G | 904A>G |
|  | R371H | 1112G>A |
| ALK | A1234T | 3700G>A |
|  | D1091N | 3271G>A |
|  | F1174C | 3521T>G |
|  | F1174I | 3520T>A |
|  | F1174L | 3522C>A |
|  | F1174V | 3520T>G |
|  | F1245C | 3734T>G |
|  | F1245V | 3733T>G |
|  | I1171N | 3512T>A |
|  | I1250T | 3749T>C |
|  | M1166R | 3497T>G |
|  | R1275Q | 3824G>A |
|  | V1135E | 3404T>A |
| BRAF | G464R | 1390G>C |
|  | G464V | 1391G>T |
|  | G466A | 1397G>C |
|  | G466R | 1396G>C |
|  | F468C | 1403T>G |
|  | G469R | 1405G>C |
|  | G469R | 1405G>A |
|  | G469S | 1405_1407GGA>AGC |
|  | G464E | 1391G>A |
|  | G466V | 1397G>T |
|  | G466E | 1397G>A |
|  | G469A | 1406G>C |
|  | G469E | 1406G>A |
|  | G469V | 1406G>T |
|  | F595L | 1785T>G |
|  | G596R | 1786G>C |
|  | L597V | 1789C>G |
|  | L597S | C1789_1790CT>TCG |
|  | V600E | 1799T>A |
|  | V600K | 1798_1799GT>AA |
|  | V600R | 1798_1799GT>AG |
|  | K601N | 1803A>T |
|  | K601E | 1801A>G |
|  | D594G | 1781A>G |
|  | D594V | 1781A>T |
|  | L597R | 1790T>G |
|  | L597Q | 1790T>A |
|  | T599I | 1796C>T |
|  | V600L | 1798G>C |
| CDK4 | R24C | 70C>T |
|  | R24H | 71G>A |
| DDR2 | I120M | 360C>G |
|  | L63V | 187C>G |
|  | L239R | 716T>A |
|  | G253C | 757G>T |
|  | G505A | 1514G>C |
|  | C580Y | 1739G>A |
|  | I638F | 1912A>T |
|  | T765P | 2293A>C |
|  | G774E | 2321G>A |
|  | G774V | 2321G>T |
| EGFR | R108K | 323G>A |
|  | T263P | 787A>C |
|  | A289V | 866C>T |
|  | G598V | 1793G>T |
|  | E746_A750del_1A | 2235_2249delGGAATTAAGAGAAGC |
|  | E746_A750del_1B | 2236_2250delGAATTAAGAGAAGCA |
|  | S752_I759del | 2254_2277delTCTCCGAAAGCCAACAAGGAAATC |
|  | L747_E749del, A750P | 2239_2247delTTAAGAGAA,2248G>C |
|  | L747_E749del, A750P | 2239_2247delTTAAGAGAA,2248G>C |
|  | L747_S752del, P753S | 2240_2257delTAAGAGAAGCAACATCTC |
|  | L747_T751del | 2240_2254delTAAGAGAAGCAACAT |
|  | L747_T750del, P ins | 2239_2250delTTAAGAGAAGCA,2251A>C |
|  | L747_T750del, P ins | 2239_2250delTTAAGAGAAGCA,2251A>C |
|  | E746_T751del, V ins | 2237_2254delAATTAAGAGAAGCAACAT,2255C>T |
|  | E746_T751del, V ins | 2237_2254delAATTAAGAGAAGCAACAT,2255C>T |
|  | L747_S752del | 2239_2256delTTAAGAGAAGCAACATCT |
|  | E746_T751del, I ins | 2235_2252delGGAATTAAGAGAAGCAAC,2254T>A,2255C>T |
|  | E746_T751del, I ins | 2235_2252delGGAATTAAGAGAAGCAAC,2254T>A,2255C>T |
|  | E746_A750del, V ins | 2237_2251delAATTAAGAGAAGCAA,2252C>T |
|  | E746_A750del, V ins | 2237_2251delAATTAAGAGAAGCAA,2252C>T |
|  | L747_S752del, Q ins | 2239_2256delTTAAGAGAAGCAACATCT,2258C>A |
|  | L747_S752del, Q ins | 2239_2256delTTAAGAGAAGCAACATCT,2258C>A |
|  | E746_A750del, T751A | 2235_2249delGGAATTAAGAGAAGC,A2251G |
|  | E746_A750del, T751A | 2235_2249delGGAATTAAGAGAAGC,A2251G |
|  | E746_A750del, T751A | 2237_2251delAATTAAGAGAAGCAA |
|  | E746_T751del, S752A | 2237_2254delAATTAAGAGAAGCAACAT |
|  | E746_T751del, S752D | 2238_2255delATTAAGAGAAGCAACATC |
|  | L747_E749del | 2239_2247delTTAAGAGAA |
|  | L747_T751del | 2239_2253delTTAAGAGAAGCAACA |
|  | V769_D770insASV | 2307_2308insGCCAGCGTG |
|  | H773_V774insH | 2319_2320insCAC |
|  | D770_N771insG | 2310_2311insGGT |
|  | H773>NPY | 2316_2317insAACCCC,2317C>T |
|  | H773_V774insPH | 2319_2320insCCCCAC |
|  | H773_V774insNPH | 2319_2320insAACCCCCAC |
|  | V774_C775insHV | 2322_2323insCACGTG,2322G>C |
|  | P772_H773insV | 2315_2316insGGT |
|  | M766_A767insAI | 2298_2299insGCCATA |
|  | V769_D770insASV | 2309_2310AC>CCAGCGTGGAT |
|  | D770_N771insG | 2310_2311insGGC |
|  | D770_N771insN | 2310_2311insAAC |
|  | D770_N771>AGG | 2309_2312ACAA>CTGGTGG |
|  | H773>NPY | 2317_2317C>AACCCCT |
|  | L861Q | 2582T>A |
|  | S768I | 2303G>T |
|  | T790M | 2369C>T |
|  | L858R | 2573T>G |
|  | E709K | 2125G>A |
|  | G719A | 2156G>C |
|  | G719C | 2155G>T |
|  | G719S | 2155G>A |
|  | E709A | 2126A>C |
|  | E709G | 2126A>G |
|  | E709V | 2126A>T |
|  | E709H | 2125_2127GAA>CAT |
| EPHA3 | A435S | 1303G>T |
|  | D446Y | 1336G>T |
|  | D678E | 2034C>A |
|  | G187R | 559G>C |
|  | G518L | 1552_1553GG>TT |
|  | G766E | 2297G>A |
|  | K761N | 2283G>T |
|  | M269I | 807G>A |
|  | N379K | 1137T>G |
|  | R728L | 2183G>T |
|  | S229Y | 686C>A |
|  | S449F | 1346C>T |
|  | T166N | 497C>A |
|  | T166N | 497C>A |
|  | T393K | 1178C>A |
|  | W250R | 748T>A |
| EPHA5 | D493Y | 1477C>A |
|  | M1034I | 3102C>T |
|  | P1036A | 3106G>C |
|  | R1007Q | 3020C>T |
|  | S566Y | 1697G>T |
|  | S810I | 2429C>A |
| ERBB2 | L755P | G2263_2264TT>CCA |
|  | G776S | 2326G>A |
|  | G776LC | 2326_2326G>CTTT |
|  | G776VC | 2326_2327insTTT |
|  | G776VC | 2326_2327insTGT |
|  | M774_A775insYVMA | 2324_2325insATACGTGATGGC |
|  | P780_Y781insGSP | 2339_2340insGGGCTCCCC |
|  | S779_P780insVGS | 2335_2336insCTGTGGGCT |
|  | C311R | 931T>C |
|  | C334S | 1000T>A |
|  | D326G | 977A>G |
|  | E321G | 962A>G |
|  | L49H | 146T>A |
|  | M774_A775insAYVM | 2322_2322insGCATACGTGATG |
|  | N319D | 955A>G |
|  | S310F | 929C>T |
|  | T216S | 646A>T |
|  | T216S | 646A>T |
|  | V750E | 2249T>A |
|  | V777A | 2330T>C |
|  | W906* | 2718G>A |
|  | W906* | 2718G>A |
| ERBB4 | D595V | 1784T>A |
|  | D931Y | 2791C>A |
|  | H618P | 1853T>G |
|  | K935I | 2804T>A |
|  | N181S | 542T>C |
|  | R306S | 916G>T |
|  | T244R | 731G>C |
|  | V348L | 1042G>T |
|  | Y285C | 854T>C |
| FGFR1 | S125L | 374C>T |
|  | P252T | 754C>A |
|  | V664L | 1990G>T |
| FGFR2 | C382R | 1144T>C |
|  | D283N | 847G>A |
|  | G272V | 815G>T |
|  | H544Q | 1632C>A |
|  | N211I | 632A>T |
|  | N211I | 632A>T |
|  | N549K | 1647T>A |
|  | P253R | 758C>G |
|  | Q212K | 634C>A |
|  | R203C | 607C>T |
|  | R496T | 1487G>C |
|  | S252W | 755C>G |
|  | W290C | 870G>C |
|  | W290C | 870G>C |
|  | Y375C | 1124A>G |
| FGFR3 | R248C | 742C>T |
|  | S249C | 746C>G |
|  | G370C | 1108G>T |
|  | Y373C | 1118A>G |
|  | A391E | 1172C>A |
|  | K650E | 1948A>G |
|  | K650M | 1949A>T |
|  | K650Q | 1948A>C |
|  | K650T | 1949A>C |
|  | C228R | 682T>C |
|  | T79S | 235A>T |
| FGFR4 | A729G | 2186C>G |
|  | E681K | 2041G>A |
|  | H192fs | 576delT??? |
|  | P672T | 2014C>A |
|  | R183S | 547C>A |
|  | R411fs | 1230_1240delCCGCTTCCCTC |
|  | R616G | 1846C>G |
|  | S232I | 695G>T |
|  | S232I | 695G>T |
|  | S732N | 2195G>A |
|  | V510M | 1528G>A |
| FLT3 | I836del | 2506_2508delATC |
|  | D835E | 2505T>G |
|  | D835H | 2503G>C |
|  | D835V | 2504A>T |
|  | D835Y | 2503G>T |
| HRAS | G12V | 35G>T |
|  | G12D | 35G>A |
|  | G12S | 34G>A |
|  | G12R | 34G>C |
|  | G12C | 34G>T |
|  | G13C | 37G>T |
|  | G13D | 38G>A |
|  | G13R | 37G>C |
|  | G13S | 37G>A |
|  | G13V | 38G>T |
|  | Q61H | 183G>C |
|  | Q61P | 182A>C |
|  | Q61H | 183G>T |
|  | Q61L | 182A>T |
|  | Q61R | 182A>G |
|  | Q61K | 181C>A |
| JAK2 | V617F | 1849G>T |
| KDR | A248G | 743C>G |
|  | A973S | 2917C>A |
|  | G1145E | 3434C>T |
|  | G1308* | 3922C>A |
|  | L1140M | 3418G>T |
|  | P1210L | 3629G>A |
|  | Q2R | 5A>G |
|  | S984T | 2951C>G |
| KIT | D52N | 154G>A |
|  | Y503_F504insAY | 1509_1510insGCCTAT |
|  | W557G | 1669T>G |
|  | W557R | 1669T>A |
|  | W557R | 1669T>C |
|  | V559A | 1676T>C |
|  | V559D | 1676T>A |
|  | V559G | 1676T>G |
|  | V559I | 1675G>A |
|  | V560D | 1679T>A |
|  | V560G | 1679T>G |
|  | K550_K558del | 1648_1674delAAACCCATGTATGAAGTACAGTGGAAG |
|  | P551_V555del | 1651_1665delCCCATGTATGAAGTA |
|  | Y553_Q556del | 1657_1668delTATGAAGTACAG |
|  | K558_V560del | 1672_1680delAAGGTTGTT |
|  | K558_E562del | 1672_1686delAAGGTTGTTGAGGAG |
|  | V559del | 1675_1677delGTT |
|  | V559_V560del | 1675_1680delGTTGTT |
|  | V560del | 1678_1680delGTT |
|  | Y570_L576del | 1708_1728delTACATAGACCCAACACAACTT |
|  | E561K | 1681G>A |
|  | L576P | 1727T>C |
|  | P585P | 1755C>T |
|  | D579del | 1735_1737delGAT |
|  | K642E | 1924A>G |
|  | D816V | 2447A>T |
|  | D816Y | 2446G>T |
|  | D816H | 2446G>C |
|  | V825A | 2474T>C |
|  | E839K | 2515G>A |
|  | M552L | 1654A>C |
|  | Y568D | 1702T>G |
|  | F584S | 1751T>C |
|  | 554_559delEVQWKV | 1660_1678delGAAGTACAGTGGAAGGTT |
|  | A829P | 2485G>C |
|  | K558N | 1674G>C |
|  | N566D | 1696A>G |
|  | V569G | 1706T>G |
|  | Y553N | 1657T>A |
|  | Y823D | 2467T>G |
|  | N567K | 1701T>A |
|  | N495I | 1484A>T |
| KRAS | G12V | 35G>T |
|  | G12S | 34G>A |
|  | G12A | 35G>C |
|  | G12D | 35G>A |
|  | G12C | 34G>T |
|  | G12R | 34G>C |
|  | G12F | 34_35GG>TT |
|  | G13C | 37G>T |
|  | G13D | 38G>A |
|  | G13S | 37G>A |
|  | G13V | 38G>T |
|  | A59T | 175G>A |
|  | Q61E | 181C>G |
|  | Q61H | 183A>T |
|  | Q61H | 183A>C |
|  | Q61K | 181C>A |
|  | Q61L | 182A>T |
|  | Q61R | 182A>G |
|  | Q61P | 182A>C |
| MDM2 | SNP309 | SN309T>G |
| NRAS | G12A | 35G>C |
|  | G12C | 34G>T |
|  | G12D | 35G>A |
|  | G12S | 34G>A |
|  | G12V | 35G>T |
|  | G12R | 34G>C |
|  | G13V | 38G>T |
|  | G13R | 37G>C |
|  | G13D | 38G>A |
|  | G13C | 37G>T |
|  | G13A | 38G>C |
|  | A18T | 52G>A |
|  | Q61P | 182A>C |
|  | Q61H | 183A>T |
|  | Q61L | 182A>T |
|  | Q61R | 182A>G |
|  | Q61K | 181C>A |
|  | Q61E | 181C>G |
| NTRK1 | A107V | 320C>T |
|  | D776E | 2328T>G |
|  | G368C | 1102G>T |
|  | M375I | 1125G>T |
|  | Q80* | 238C>T |
|  | R119H | 356G>A |
|  | S326R | 978C>G |
|  | V422L | 1264G>T |
| NTRK3 | H677Y | 2029C>T |
|  | I783N | 2348A>T |
|  | L152I | 454G>T |
|  | L248M | 742G>T |
|  | L270M | 808G>T |
|  | R678Q | 2033G>A |
|  | R721F | 2161_2162CG>TT |
|  | S184C | 550T>A |
|  | S184C | 550T>A |
|  | T283K | 848G>T |
| PDGFRA | V561D | 1682T>A |
|  | T674I | 2021C>T |
|  | F808L | 2422T>C |
|  | D846Y | 2536G>T |
|  | N870S | 2609A>G |
|  | D1071N | 3211G>A |
|  | D842_H845del | 2524_2535delGACATCATGCAT |
|  | I843_D846del | 2527_2538delATCATGCATGAT |
|  | S566_E571>K | 1697_1711delGCCCAGATGGACATG |
|  | I843_S847>T | 2528_2539delTCATGCATGATT |
|  | D842V | 2525A>T |
|  | A210V | 629C>T |
|  | A210V | 629C>T |
|  | D480E | 1440C>G |
|  | E338Q | 1012G>C |
|  | G829R | 2485G>A |
|  | L580M | 1738C>A |
|  | M133K | 398T>A |
|  | V193I | 577G>A |
|  | W549* | 1647G>A |
| PIK3CA | R88Q | 263G>A |
|  | N345K | 1035T>A |
|  | C420R | 1258T>C |
|  | P539R | 1616C>G |
|  | E542K | 1624G>A |
|  | E545K | 1633G>A |
|  | Q546K | 1636C>A |
|  | H701P | 2102A>C |
|  | H1047L | 3140A>T |
|  | H1047R | 3140A>G |
|  | H1047Y | 3139C>T |
|  | G1049R | 3145G>C |
|  | R38H | 113G>A |
|  | C901F | 2702G>T |
|  | M1043I | 3129G>T |
|  | M1043V | 3127A>G |
| RET | C634R | 1900T>C |
|  | C634W | 1902C>G |
|  | C634Y | 1901G>A |
|  | E632_L633del | 1894_1899delGAGCTG |
|  | M918T | 2753T>C |
|  | A664D | 1991C>A |
